# Sudden sensorineural olfactory loss: a structured narrative review and proposal for a standardised terminological framework

**DOI:** 10.3389/fsurg.2026.1838659

**Published:** 2026-05-14

**Authors:** Mohammad H. Al-Bar

**Affiliations:** Department of Otolaryngology – Head and Neck Surgery, College of Medicine, Imam Abdulrahman Bin Faisal University, Dammam, Saudi Arabia

**Keywords:** anosmia, corticosteroids, COVID-19, ICD-11, olfactory dysfunction, olfactory training, post-viral anosmia, sensorineural smell loss

## Abstract

**Background:**

Sudden loss of smell — particularly when sensorineural in origin — is a clinically disabling condition that the medical community has never adequately named. Patients presenting with acute olfactory loss encounter a field without shared definitions, dedicated ICD codes, or guidelines built around their specific presentation. The consequence is diagnostic delay, terminological inconsistency in the literature, and a research base that cannot be meaningfully synthesised.

**Objective:**

This paper reviews the evidence on sudden sensorineural olfactory loss, demonstrates that existing terminology is inadequate, and proposes a single new clinical term: Sudden Sensorineural Olfactory Loss (SSNOL). Two descriptive aetiological categories are introduced solely to guide future research, not as additional terminology.

**Methods:**

A structured narrative review was conducted using PubMed, Embase, and Scopus databases. Studies were selected based on relevance to sudden olfactory dysfunction, neural pathway mechanisms, diagnostic imaging, and clinical management. Both primary research articles and systematic reviews published between 2000 and 2024 were considered.

**Results:**

The evidence describes a condition with serious, sometimes permanent neural consequences. Post-viral olfactory loss — which received relatively limited specialist attention before the COVID-19 pandemic — was suddenly experienced by hundreds of millions of people, exposing how poorly the field was equipped to respond. Diagnostic tools are blunt. Treatment options are thin. Olfactory training is the only intervention with consistent evidence behind it. None of this will improve without better terminology to organise research around.

**Conclusion:**

We propose the term “Sudden Sensorineural Olfactory Loss” (SSNOL) as the sole terminological contribution of this paper, modelled on the established precedent of sudden sensorineural hearing loss (SSNHL). To guide future research, two descriptive aetiological categories are described — SSNOL-Viral and SSNOL-Idiopathic — presented as hypothesis-generating models to support future research design. Traumatic olfactory loss is explicitly excluded from the SSNOL definition and should be managed under existing post-traumatic frameworks.

## 1 Introduction

Sudden loss of the sense of smell is a clinically significant and increasingly recognised presentation that the medical community has never adequately named. Olfaction serves critical functions in environmental hazard detection, nutrition, and emotional processing (1–3), and its abrupt loss carries substantial psychosocial consequences that clinicians routinely underestimate. Olfactory dysfunction, ranging from partial impairment (hyposmia) to complete loss (anosmia), affects an estimated 5% of the general population, with rates rising steeply among older adults and after viral illness (1, 2). Despite this prevalence, the field lacks a unified clinical language, shared case definitions, or dedicated treatment guidelines for the acute sensorineural variant. The COVID-19 pandemic transformed this long-standing terminological gap from an academic concern into a global clinical problem.

COVID-19 changed this. Anosmia affected 34%–98% of infected individuals in early-pandemic cohorts (4, 5) — tens of millions of people lost their sense of smell simultaneously, and a field that had not prioritised this problem was thrust into scrutiny. What that exposure revealed was damaging: terms including “anosmia”, “post-viral olfactory disorder”, “olfactory nerve damage”, and “sensorineural smell loss” were being used interchangeably across journals (6, 7). No shared case definition. No consistent diagnostic criteria. Researchers could not meaningfully compare their findings because they were not, in any coherent clinical sense, studying the same entity.

The mechanistic anatomy is not complicated. Olfactory loss is conductive when airflow to the epithelium is blocked, sensorineural when the receptor neurons or nerve are damaged, and central when the olfactory bulb or cortical pathways are involved (8). In most acute presentations the pathology is principally sensorineural. The hearing loss field recognised this distinction decades ago and built SSNHL around it. Olfaction has not followed. The result is a category of patients — those with sudden sensorineural smell loss — who cannot be named, coded, or meaningfully enrolled in clinical trials (9).

This paper addresses this gap through three aims: (1) to review the evidence on sudden sensorineural olfactory loss; (2) to make the case that existing terminology is inadequate; and (3) to propose one new clinical term — SSNOL — modelled on the SSNHL precedent. A classification diagram, comparative terminology table, and ICD-11 integration roadmap accompany the proposal.

## 2 Methods

This is a structured narrative review. Its purpose is synthesis and argument, not statistical pooling.

A literature search was performed using PubMed/MEDLINE, Embase, and Scopus databases covering the period from January 2000 to December 2024. Search terms included combinations of: “anosmia”, “hyposmia”, “olfactory dysfunction”, “sudden olfactory loss”, “sensorineural”, “post-viral”, “COVID-19”, “olfactory bulb”, “olfactory epithelium”, and “olfactory training”. Studies were selected based on relevance to mechanisms, epidemiology, diagnosis, or treatment of acute or sudden olfactory dysfunction. Priority was given to systematic reviews, randomised controlled trials, and large cohort studies where available, supplemented by key mechanistic and imaging studies.

Given the conceptual and integrative aim of this review, formal meta-analysis and quantitative synthesis were not performed. Findings were synthesised thematically, with emphasis on clinical applicability and identification of gaps in current terminology and classification. The initial search yielded approximately 1,200 records. Title and abstract screening applied four explicit exclusion filters: (i) studies not addressing olfaction or addressing only peripheral sinonasal obstruction without a sensorineural component; (ii) non-English publications; (iii) conference abstracts lacking full-text data; and (iv) cross-database duplicates. Approximately 180 full-text articles passed this stage and were reviewed against the inclusion criteria detailed below. Of these, 48 articles were selected for citation based on direct relevance to terminology, mechanism, epidemiology, or management of sudden sensorineural olfactory loss, with priority given to systematic reviews, position papers, and landmark mechanistic studies.

Inclusion criteria required that studies: (1) reported outcomes related to olfactory function or dysfunction; (2) addressed mechanism, epidemiology, diagnosis, or treatment of sudden or acute olfactory loss; and (3) were available in English. Case reports were included only when they described novel mechanistic findings. This review follows a structured narrative methodology rather than PRISMA systematic review methodology, consistent with its conceptual and terminological analysis objectives.

## 3 Literature review

### 3.1 Anatomy and physiology of the olfactory system

The peripheral olfactory apparatus consists of approximately six million bipolar olfactory receptor neurons (ORNs) distributed across the olfactory epithelium, which lines the roof of the nasal cavity and portions of the superior turbinate and nasal septum (10). The ORNs express odorant receptor proteins encoded by the largest gene family in the human genome — comprising approximately 400 functional olfactory receptor genes alongside a similar number of pseudogenes — enabling discrimination of thousands of distinct odorants (11). Odorant molecules trigger a G-protein-coupled signalling cascade generating action potentials transmitted via the olfactory nerve (CN I) to the olfactory bulb, and thence to piriform cortex, amygdala, entorhinal cortex, and orbitofrontal cortex (12, 13).

One feature of the olfactory system is genuinely remarkable: unlike most neural tissue, ORNs regenerate. Their normal lifespan is 30–60 days, with continuous replacement from basal stem cells (14). This is why recovery from peripheral olfactory loss is possible at all. But regeneration fails when it is most needed. Sustained inflammation, mucosal scarring, and basal cell depletion — the very consequences of severe viral infection and trauma — disrupt the process entirely (15).

### 3.2 Epidemiology of olfactory dysfunction

Pre-pandemic estimates placed clinically significant olfactory dysfunction at 3%–20% of the population (1). COVID-19 altered the epidemiological landscape substantially: a systematic review by Tong et al. (4) estimated dysfunction in 47% of COVID-19 cases overall, with early-pandemic cohorts exceeding 80%. Persistent loss beyond 12 weeks affected 10%–30% of patients (16). Among specialist clinic referrals, the breakdown before COVID was: sinonasal disease (20%–30%), post-viral causes (18%–45%), head trauma (7%–18%), and idiopathic presentations (18%–25%) (3). Post-viral is now the dominant category by a substantial margin.

### 3.3 Mechanisms of sudden sensorineural olfactory loss

#### 3.3.1 Post-Viral mechanisms

Single-cell RNA sequencing provided a counterintuitive insight: SARS-CoV-2 does not primarily infect olfactory receptor neurons at all. Sustentacular support cells — which lack the neural specialisation of ORNs but express high levels of ACE2 and TMPRSS2 — are the principal viral entry point (17). Their destruction is sufficient to functionally incapacitate ORNs through local inflammation, oedema, and ciliary loss. In cases of persistent dysfunction, the damage extends further: ongoing neuroinflammation, olfactory bulb volume reduction, and cortical structural changes have all been documented (15, 18). Sustained sensory deprivation drives cross-modal cortical reorganisation, with olfactory cortex progressively recruited to non-olfactory tasks (19). This plasticity may not reverse even when peripheral input is restored — which is precisely why early diagnosis matters, and why the absence of agreed diagnostic criteria has real clinical consequences.

#### 3.3.2 Traumatic olfactory loss — exclusion from the SSNOL definition

Head trauma causes anosmia in 4%–16% of traumatic brain injury cases (20). The mechanism is distinct: shear forces at the cribriform plate damage CN I axons directly, with prognosis governed by bulb integrity on MRI rather than by the post-viral neuroregenerative biology that underpins non-traumatic SSNOL (21, 22). This mechanistic divergence justifies explicit exclusion of traumatic olfactory loss from the SSNOL definition — directly mirroring the SSNHL convention in which barotrauma and blast-related hearing loss are managed as separate clinical entities. Clinicians must elicit a clear trauma history before applying SSNOL criteria; a positive history redirects to post-traumatic olfactory loss pathways.

A clarification is warranted for mild TBI presentations. Olfactory loss can follow even mild head trauma without radiographic fracture, as shearing forces at the cribriform plate may damage olfactory nerve fibres at injury magnitudes below the threshold for detectable bony injury (23). Any documented head trauma temporally associated with olfactory loss — regardless of severity classification or imaging findings — triggers the SSNOL exclusion criterion and redirects management to post-traumatic olfactory loss pathways. This conservative boundary mirrors the SSNHL approach of separating trauma-related cases from idiopathic sensorineural loss and avoids mechanistic dilution of the SSNOL category.

#### 3.3.3 Idiopathic sudden olfactory loss

Idiopathic sudden olfactory loss — characterised by acute onset without identifiable cause — may represent a heterogeneous syndrome encompassing microvascular ischaemia, autoimmune attack, or subclinical viral infection (24). The absence of a universally accepted case definition has hampered epidemiological study. Diagnostic delays are well documented: the Refsum disease paradigm illustrates how poorly characterised olfactory presentations can result in decades-long diagnostic odysseys (25).

#### 3.3.4 Neural reorganisation and central consequences

SSNOL is not solely a peripheral sensory problem. Progressive olfactory bulb atrophy (26) and cross-modal cortical reorganisation (19) confirm that untreated peripheral loss drives structural brain-level consequences. Some of these changes may not reverse when peripheral function recovers. This is relevant to prognosis, to the timing of intervention, and to why delayed diagnosis — which the current terminological vacuum actively promotes — has measurable clinical cost.

The magnitude and reversibility of this plasticity are not uniform across the lifespan. During early postnatal development, sensory cortices undergo critical-period maturation in which functional specialisation is tightly coupled to ongoing peripheral input — sensory deprivation during these windows produces lasting alterations in cortical organisation that differ mechanistically from adult-acquired reorganisation (27, 28). Recent work has further characterised how peripheral sensory input shapes cortical area identity at the cell-type level during development (29), while the broader framework of hierarchical critical-period plasticity across the sensorimotor-association axis has been synthesised in detail (30). Cross-modal effects in adulthood — relevant to acquired olfactory loss — typically involve reorganisation of already-mature circuits rather than failure of their initial formation. This life-stage dependence has two implications for the SSNOL proposal. First, the clinical-urgency argument advanced in this paper rests on adult-acquired plasticity rather than developmental plasticity, and is therefore most directly applicable to adolescent and adult presentations. Second, paediatric SSNOL — already flagged in our Limitations section as warranting dedicated future work — may carry distinct mechanistic and prognostic considerations that justify separate sub-classification.

### 3.4 Alterations in the olfactory epithelium and receptor neurons

Biopsy evidence from post-viral olfactory specimens is striking: ciliary loss, epithelial denudation, inflammatory infiltration, and patchy replacement of receptor cells by non-sensory epithelium (15, 31). At the molecular level, reduced OMP immunoreactivity tracks functional impairment and may in time serve as a tissue biomarker for disease staging (32). The histological profile of this condition is mechanistically distinct from conductive or central olfactory loss, and supports a separate clinical category.

### 3.5 Clinical presentation and assessment

Clinically, patients are usually clear about what happened. There was a moment — often anchored to a viral illness — when smell simply went. That precision of onset is diagnostically important. Parosmia and phantosmia, which emerge during partial recovery, reflect aberrant ORN regeneration rather than new pathology (33). Objective assessment with Sniffin' Sticks (TDI), UPSIT, or CCCRC is essential (34); clinical impression alone is unreliable. MRI of the olfactory bulbs and olfactory event-related potentials (oERPs) add structural and electrophysiological grounding when the picture is unclear.

### 3.6 Current therapeutic approaches and their limitations

#### 3.6.1 Pharmacological interventions

The pharmacological evidence base is limited. Systemic corticosteroids — widely prescribed despite limited justification — lack sufficient evidence for routine use in post-infectious olfactory loss according to current clinical practice guidelines (35). Topical steroids, sodium citrate rinses, omega-3 supplementation, and alpha-lipoic acid have all been explored. None has cleared the bar for guideline recommendation (36). The field is treating a serious condition with interventions whose evidence base requires strengthening.

#### 3.6.2 Olfactory training

Olfactory training — twice-daily deliberate sniffing of four defined odorants, as described by Hummel et al. (37) — is the exception to the above. It works. Multiple RCTs across post-traumatic and post-infectious aetiologies show consistent TDI improvement (38, 39), and a meta-analysis by Pekala et al. (40) confirmed the effect is statistically robust, with duration beyond 12 weeks conferring additional benefit. A 13-year evidence synthesis reinforced its cross-aetiology efficacy (41). It is the only intervention the field currently has that warrants genuine clinical confidence.

#### 3.6.3 Emerging therapies

Omega-3 fatty acid supplementation has shown preliminary promise in post-surgical olfactory dysfunction (42), and platelet-rich plasma (PRP) injection into the olfactory cleft is an emerging approach under early investigation. Intranasal delivery approaches — exploiting the olfactory epithelium's direct CNS access — may in future enable targeted delivery of neuroprotective or regenerative agents (43). The SSNOL descriptive categories are designed to enable aetiology-specific therapeutic trials, a key gap in the current literature.

## 4 Critical evaluation of existing terminology

### 4.1 The limitations of “anosmia” and “hyposmia”

SSNOL does not replace “anosmia” or “hyposmia”. Those terms describe what the patient experiences. SSNOL describes what is happening mechanistically, and when. The distinction matters because “anosmia” currently groups patients who share almost nothing clinically. A man with congenital Kallmann syndrome, a woman with a frontal meningioma compressing the olfactory tract, and a previously healthy adult who woke up unable to smell after a viral illness all receive the same label (3). Same code. Radically different pathology, prognosis, and management. Collapsing them into one category does not serve patients or research.

### 4.2 The inadequacy of “post-viral olfactory dysfunction” and related terms

Hernandez et al. (44) recently standardised definitions for common olfactory symptom descriptors — anosmia, hyposmia, parosmia, and related terms. That work answered the question of what each symptom means. SSNOL answers a different question: what is this condition? The two proposals operate at different levels and are complementary rather than competing.

“Post-viral olfactory dysfunction” (PVOD) at least identifies aetiology. But it tells the clinician nothing about neural mechanism, acuity of onset, or whether central involvement should be suspected. “Idiopathic anosmia” is worse: it communicates the absence of a diagnosis rather than the presence of a clinical entity. Applied to a heterogeneous group who may have microvascular, autoimmune, or subclinical viral pathology undetected by current tools (24), it offers neither prognostic nor research value. Table 1 sets these terms against SSNOL across the dimensions that matter clinically.

**Table 1:** Systematic comparison of existing olfactory dysfunction terminology against the proposed SSNOL framework.

Feature	Anosmia	PVOD	SSNOL (proposed)
Onset acuity	Not specified	Not specified	Acute (≤72 h) — explicit
Mechanism specified	No	Partial (viral only)	Yes — sensorineural
Aetiology covered	All causes (non-specific)	Viral only	Viral/URTI, idiopathic (traumatic excluded)
Neural vs. conductive distinction	No	No	Yes — explicit
Prognosis guidance	None	Limited	Via descriptive categories
ICD coding utility	R43.0 (generic)	Partial	Proposed R43.0 modifiers
Research comparability	Poor	Moderate	High — standardised
Guideline applicability	None specific	Limited	Intended for guidelines
Hearing loss analogy	No analogy	No analogy	Parallels SSNHL model

PVOD, post-viral olfactory dysfunction; SSNOL, sudden sensorineural olfactory loss; ICD, International Classification of Diseases; SSNHL, sudden sensorineural hearing loss.

### 4.3 The analogy with sensorineural hearing loss

Because the SSNHL precedent is central to this proposal, a brief history of how that terminology developed is warranted. Prior to the formal definition of SSNHL in the late twentieth century, sudden hearing loss of unclear aetiology was variably described in the literature, complicating cross-study comparison and delaying the development of standardised management. The adoption of a unified mechanistic term — one that distinguished sensorineural pathology from conductive and central causes — provided the conceptual anchor around which case definitions, diagnostic criteria, and ultimately the AAO-HNS clinical practice guideline were built (45). The 2019 update of that guideline now codifies the 72-h onset criterion, recommends prompt psychophysical audiometric confirmation, and provides graded recommendations on corticosteroid use. The transformation from a poorly characterised clinical entity into a guideline-supported specialty took several decades, but the terminological step came first. The same sequential pattern is what this paper proposes for olfaction.

The analogy with hearing loss is not decorative — it is the central argument. SSNHL began as a heterogeneous, poorly-defined clinical problem. A single named term, built on mechanistic criteria, preceded everything else: the guidelines (45), the treatment protocols, the research infrastructure. Terminology was the catalyst, not the conclusion. SSNOL is proposed as the equivalent first step for olfaction.

## 5 Proposed terminological framework: SSNOL

### 5.1 Defining SSNOL as a unified clinical entity

We propose Sudden Sensorineural Olfactory Loss (SSNOL) as the sole clinical term to denote olfactory dysfunction characterised by:

(1) Onset within 72 h
(2) Sensorineural mechanism (peripheral and/or central)
(3) Absence of purely conductive causes (e.g., nasal polyps, significant septal deviation)
(4) Absence of recent head trauma (absolute exclusion criterion)

Table 2 summarises the proposed diagnostic criteria for SSNOL. The term “sensorineural” is borrowed from the hearing loss literature, where it carries a specific anatomical meaning — involvement of sensory receptor cells and/or neural pathways rather than conductive structures — understood by every otolaryngologist. Using it for olfactory loss is mechanistic alignment, not metaphor. “Loss” follows SSNHL convention: a measurable functional deficit, not merely a symptom. SSNOL is the single term this paper proposes. The descriptive aetiological categories described in Section 5.2 are research scaffolding, not co-proposed terminology. One ask of the field is achievable. Multiple simultaneous asks are not.

**Table 2:** Proposed diagnostic criteria for SSNOL.

Criterion	Specification
Onset	Within 72 h
Objective confirmation	Psychophysical testing (TDI or UPSIT) below age- and sex-adjusted norms
Exclusion of conductive causes	Clinical and endoscopic assessment
Exclusion of head trauma	Absolute criterion; positive history redirects to post-traumatic pathways
Aetiological descriptor	SSNOL-Viral or SSNOL-Idiopathic (descriptive only, for research stratification)

All criteria require prospective validation. The aetiological descriptor (criterion 5) is used descriptively for research stratification, not as a separate diagnosis.

### 5.2 Descriptive aetiological categories (not co-proposed terminology)

To prevent the terminological fragmentation this paper seeks to resolve, we explicitly reject the creation of new clinical acronyms. Instead, we describe two aetiological categories to be used descriptively under the SSNOL umbrella to guide research and prognosis:

These categories are presented as hypothesis-generating frameworks to guide future validation studies — not as co-proposed terminology requiring simultaneous adoption. The field needs one term. Once SSNOL is accepted, the descriptive categories can be validated, refined, or replaced through prospective research. These two descriptive categories are summarised in Table 3.

**Table 3:** Descriptive aetiological categories under the SSNOL framework.

Category	Description	Purpose
SSNOL-viral	Post-viral presentations (SARS-CoV-2, other respiratory viruses) involving sustentacular cell damage, epithelial inflammation, and potential central neuroinflammation	Captures the numerically largest group; identifies multifactorial pathology requiring aetiology-specific study
SSNOL-idiopathic	Diagnosis of exclusion for sudden loss with no identifiable trigger; presumed microvascular, autoimmune, or subclinical viral aetiology	Directs investigation rather than closing the diagnostic conversation; enables dedicated registry development

These are research scaffolding, not co-proposed clinical terminology.

### 5.3 Diagnostic criteria for SSNOL

A duration of at least seven days is included as a retrospective confirmatory criterion to exclude transient dysfunction, not a waiting period before assessment. Early clinical evaluation is indicated regardless; delayed presentation carries neuroplastic risk (19). All criteria require prospective validation.

Rationale for the 72-h window. The 72-h onset criterion is adopted directly from the SSNHL precedent, where it has served as the operational definition of acute sensorineural loss for decades and is codified in the 2019 AAO-HNS clinical practice guideline (45). Three considerations support transposing this threshold to olfaction. First, terminological consistency with SSNHL is a central argument of this proposal; adopting a divergent threshold would weaken the analogical framework without offering clear biological justification. Second, a 24-h window would exclude a clinically important proportion of patients who present with progressive loss over two to three days — a presentation well documented in post-viral olfactory loss. Third, extending the window beyond 72 h risks conceptual overlap with subacute and chronic post-infectious olfactory dysfunction, which the SSNOL framework is specifically designed to distinguish from. Beyond this analogical reasoning, the 72-h threshold also corresponds approximately to the early phase during which cortical reorganisation begins to emerge in the setting of sensory deprivation (Section 3.3.4) — providing an olfaction-specific biological rationale. The threshold is nevertheless explicitly identified as a candidate for empirical refinement in prospective validation studies.

The sub-acute “gray zone” (Day 4–7). Patients presenting with sudden olfactory loss that began four to seven days before evaluation fall outside the strict 72-h window but remain within the early post-onset period, during which the arguments for neuroplastic urgency (Section 3.3.4) are still biologically plausible. In clinical practice, these patients should not be categorically excluded from SSNOL-informed management. The framework accommodates them through two mechanisms: (i) if historical onset was sudden and within 72 h with evaluation merely delayed, the patient meets SSNOL criteria retrospectively; (ii) if onset itself extended over four to seven days, the patient is managed as “probable SSNOL” with conservative-leaning shared decision-making (Section 5.5), objective psychophysical confirmation, and olfactory training initiated without delay. Formal incorporation of this sub-acute category into the diagnostic criteria themselves will require prospective data and is identified as a priority for the validation phases described in Section 6.3.

### 5.4 Illustrative clinical application

To demonstrate practical utility, two illustrative clinical scenarios are presented, followed by a third case demonstrating correct exclusion. These are hypothetical composite cases.

#### 5.4.1 Case 1 — SSNOL-viral (descriptive category)

A 42-year-old female develops complete loss of smell within 48 h of confirmed SARS-CoV-2 infection, accompanied by parosmia, fatigue, and cognitive symptoms persisting beyond 12 weeks. Under current terminology she receives “post-COVID anosmia” — a label that captures aetiology but not the neural mechanism, the syndromic character, or the prognostic category. Under the SSNOL framework she meets diagnostic criteria and is described as SSNOL-Viral: multifactorial post-viral aetiology, long COVID association, moderate prognosis, and olfactory training as first-line intervention. Objective TDI confirmation within 72 h initiates the shared steroid decision-making process described in Section 5.5 (46, 47).

#### 5.4.2 Case 2 — SSNOL-idiopathic (descriptive category)

A 58-year-old male presents with sudden complete anosmia of 10 days duration with no preceding viral illness, no head trauma, and no identifiable cause on comprehensive clinical, endoscopic, laboratory, and MRI evaluation. Under current terminology he receives “idiopathic anosmia” — a label that closes the diagnostic conversation. Under the SSNOL framework he meets SSNOL criteria and is described as SSNOL-Idiopathic: exclusion diagnosis, presumed sensorineural aetiology, variable prognosis, and a profile suitable for dedicated registries and future therapeutic trials.

#### 5.4.3 Case 3 — correct exclusion (traumatic)

A 34-year-old male presents with complete anosmia within 24 h of a road traffic accident. CT imaging reveals a cribriform plate fracture and MRI confirms bilateral olfactory bulb contusion. This patient does not meet SSNOL criteria — the trauma history triggers the fourth exclusion criterion immediately. He is managed under post-traumatic olfactory loss pathways. This case demonstrates that SSNOL correctly identifies mechanistically distinct patient populations rather than conflating them.

These scenarios illustrate that SSNOL — including its explicit exclusion criteria and descriptive categories — changes the clinical narrative, directs investigation, informs prognosis, and enables aetiology-stratified research enrolment in ways that current terminology does not.

### 5.5 The steroid decision dilemma: a framework for clinical practice

The most clinically consequential question this framework raises is also the most practically difficult: should patients with sudden olfactory loss receive oral corticosteroids? The SSNHL paradigm, on which SSNOL is modelled, has established oral steroids as a first-line intervention when administered early — the 2019 AAO-HNS guideline recommends prompt corticosteroid therapy as both primary and salvage treatment for idiopathic SSNHL (45). Transposing this approach to olfactory loss is tempting but not straightforward.

The critical difference is epidemiological scale. SSNHL occurs in approximately 5–20 per 100,000 people per year. Acute olfactory loss following URTI — including but not limited to COVID-19 — affects tens of millions annually. Spontaneous recovery of olfactory function has been reported in up to 70%–80% of mild COVID-19 cases within four weeks of onset (16), and recovery rates in non-COVID URTI are similarly favourable (46). If every patient presenting with post-URTI smell loss is considered a candidate for systemic corticosteroids, the risk-benefit calculation is profoundly different from that in SSNHL: a condition with high spontaneous recovery rates, treated with an immunosuppressant carrying well-documented risks including hyperglycaemia, adrenal suppression, avascular necrosis, and neuropsychiatric effects (47).

Yet a refusal to treat is not a neutral choice. For the subset of patients who will not recover spontaneously — those who will go on to develop persistent SSNOL — the early post-onset window may be the only period during which intervention can prevent central neuroplastic entrenchment. Once cortical reorganisation is established (19), the therapeutic window may close. Not treating everyone carries its own cost: it may abandon those individuals in whom early intervention could have made a difference.

The SSNOL framework enables a stratified, evidence-informed approach:

Without objective confirmation: Patients reporting smell loss without psychophysical confirmation — which accounts for the majority of URTI-associated olfactory complaints — should be managed conservatively: watchful waiting, early olfactory training, and reassessment at four weeks. The probability of spontaneous resolution in this group is high (46).
With objective confirmation (SSNOL criteria met within 72 h): The calculus changes. This patient has confirmed sensorineural loss in the early post-onset period — the population in whom an SSNHL-informed steroid trial is at least biologically plausible. A clinical discussion of a short course of oral corticosteroids — with an explicit informed-consent discussion of the limited evidence base for olfactory loss specifically (35, 48) and the established safety profile in short-course use (47) — mirrors the shared decision-making model recommended for idiopathic SSNHL (45) and is defensible within the SSNOL framework.

This is not a recommendation to treat; it is a recommendation to have the conversation, documented, with objective evidence in hand. This stratification is exactly what standardised criteria enable. Without SSNOL — without objective confirmation as a required criterion, without a defined 72-h window, without an explicit distinction from self-limiting URTI smell loss — there is no rational basis on which to decide who to treat. The clinical problem of who should receive steroids is inseparable from the terminological problem of who has SSNOL.

## 6 Roadmap for ICD-11 integration and international guideline adoption

### 6.1 Rationale for coding integration

ICD-10's R43.0 aggregates conductive, sensorineural, and central olfactory loss under a single entry, rendering disease burden estimates unreliable and international comparisons meaningless. ICD-11's CA0A improves on this only marginally. Until olfactory loss subtypes receive distinct codes, health systems cannot plan for them, researchers cannot aggregate across them, and patients cannot access condition-specific clinical pathways.

Integration of the SSNOL framework into ICD-11 could represent a meaningful advance if validated and adopted: for the first time, clinicians could assign mechanistically meaningful codes that distinguish viral/URTI presentations (SSNOL-Viral) from idiopathic cases (SSNOL-Idiopathic), with traumatic olfactory loss coded under existing post-traumatic frameworks.

### 6.2 Proposed ICD-11 coding structure

Table 4 outlines the proposed coding structure. Crucially, only SSNOL is proposed as a formal diagnostic code. The modifiers are presented as optional granularity for research and epidemiological surveillance, not as separate diagnostic entities.

**Table 4:** Proposed ICD-11 coding structure for the SSNOL framework.

Diagnosis	Proposed ICD-11 Code	Target Guideline Body	Priority
SSNOL (unified entity)	CA0A.0Z — Olfactory dysfunction, NOS (modified)	WHO/ICD-11 committee	High
SSNOL-Viral (descriptive modifier)	CA0A.02 modifier	ERS, WHO COVID task force	Urgent
SSNOL-Idiopathic (descriptive modifier)	CA0A.03 modifier	AChemS, ERS	Moderate

ARS, American Rhinologic Society; ERS, European Rhinologic Society; AAO-HNS, American Academy of Otolaryngology — Head and Neck Surgery; AChemS, Association for Chemoreception Sciences; NOS, not otherwise specified; WHO, World Health Organisation.

### 6.3 Pathway to international guideline adoption

#### 6.3.1 Phase 1 — terminological consensus

Formal endorsement by the ERS and ARS through a joint position statement, with AChemS engagement to anchor the term in chemosensory science. Without this, nothing else follows.

To move from a theoretical proposal to clinical adoption, a formal consensus process using Delphi methodology is warranted. This should involve a multidisciplinary panel of rhinologists, neuro-otologists, sensory scientists, and primary-care representatives to refine the diagnostic criteria, severity grading, and operational thresholds of SSNOL. A structured Delphi process would systematically aggregate expert judgement, identify points of divergence, and produce defensible operational definitions in advance of formal society endorsement.

#### 6.3.2 Phase 2 — prospective clinical validation

Multicentre cohort studies applying the SSNOL diagnostic criteria to prospectively enrolled patients, with descriptive category-stratified outcome reporting. This evidence base will demonstrate clinical utility and identify criteria requiring refinement.

#### 6.3.3 Phase 3 — formal guideline development

Structured expert panel processes (e.g., GRADE methodology) producing evidence-based management recommendations for SSNOL, with guidance stratified by descriptive category.

#### 6.3.4 Phase 4 — ICD integration

A formal application to the ICD-11 Maintenance Platform with supporting evidence from phases 1–3.

#### 6.3.5 Phase 5 — SNOMED CT integration

Enabling interoperability across electronic health record systems globally.

### 6.4 Implications for clinical practice and research

For the clinician, SSNOL classification changes the consultation. A patient described as SSNOL-Viral following COVID-19 needs a multidisciplinary long COVID pathway and an objective-testing-gated discussion of early steroid therapy — not blanket reassurance. A patient described as SSNOL-Idiopathic needs enrolment in a registry and a clear explanation that their exclusion diagnosis is the beginning of a clinical investigation, not the end. A patient presenting after head trauma must be redirected to post-traumatic pathways before SSNOL criteria are considered at all.

For patients, standardised terminology reduces diagnostic delay — currently a major source of distress — and improves access to appropriate specialist care. For the research community, standardised codes enable large-scale registry development, biobank linkage, and ultimately the powered RCTs needed to establish evidence-based treatments stratified by SSNOL descriptive category, including definitive steroid trials.

## 7 Discussion

Sudden olfactory loss of sensorineural origin affects a large number of patients, causes serious harm, and generates a body of literature that cannot be synthesised because its basic terminology is inconsistent. SSNOL does not resolve the underlying science. It creates the lexical infrastructure that better science requires.

COVID-19 proved a turning point for olfactory medicine, generating research that revealed both the field's potential and its foundational limitations. The terminological fragmentation that pre-existed the pandemic persists, making cross-study comparison difficult. The SSNOL framework is designed to address this by providing a common language that can be adopted globally without requiring fundamental revision as the evidence base evolves.

SSNHL is the proof of concept. It was not always a well-defined clinical entity with its own AAO-HNS guideline (45) and established treatment algorithms. It became one through exactly the process proposed here: a named term, criteria, professional society endorsement, and the research infrastructure that followed. The same sequence offers a credible path for olfaction, subject to field-specific adaptation.

Several limitations warrant acknowledgement. The 72-h onset criterion is a working choice, not a validated threshold. The boundary between SSNOL-Viral and SSNOL-Idiopathic will be difficult to draw in cases of suspected but unconfirmed viral aetiology. Paediatric presentations and neurodegenerative-associated olfactory loss are not addressed and require dedicated sub-classification work. The steroid decision framework, while clinically reasoned, awaits prospective validation. None of these limitations invalidates the core proposal. They are identified precisely so that validation studies can address them.

The single-term focus of this proposal is deliberate. The field does not need multiple new acronyms simultaneously. It needs one well-defined, mechanistically grounded term around which consensus can form. The descriptive categories presented here are starting points for research, not co-proposals. If SSNOL gains acceptance, the categories can be validated, refined, or replaced based on evidence.

## 8 Conclusion

SSNOL is a single, anatomically grounded, mechanistically specific clinical term for sudden olfactory loss of sensorineural origin, modelled on the SSNHL precedent that has served otology well for decades. It is the first thing the field needs and currently lacks. The descriptive aetiological categories described alongside it — SSNOL-Viral and SSNOL-Idiopathic — are research scaffolds, not co-proposals. They are starting points for the sub-classification work that prospective validation and expert consensus must determine.

This framework offers a structured basis for prospective clinical evaluation. The roadmap for ICD-11 integration is laid out. The analogy with hearing loss is not merely suggestive — it is a worked example of what terminological standardisation can achieve. Olfaction deserves the same.

## 9 Limitations

This work has several important limitations. First, it represents a structured narrative review rather than a systematic review or meta-analysis, and is therefore subject to potential selection bias. Second, the proposed SSNOL framework has not yet been validated in prospective clinical cohorts or through formal expert consensus methodologies such as Delphi processes. Third, the descriptive categories may exhibit clinical overlap in certain presentations — particularly in cases where viral aetiology is suspected but unconfirmed, or where exclusion of conductive causes is incomplete. Fourth, this is a single-author proposal, and the absence of a multidisciplinary authorship panel may limit the representativeness of the perspectives incorporated.

Fifth, the formalisation of SSNOL as a distinct clinical entity carries risks that validation studies must address: over-medicalisation of transient self-limited anosmia, potential over-prescription of corticosteroids in the early post-onset window before evidence-based protocols are established, and transitional coding confusion as ICD-11 integration unfolds. The framework's purpose is organisational — reducing diagnostic heterogeneity and enabling stratified research — rather than expanding the scope of diagnosis. Validation studies should incorporate clear criteria for distinguishing cases that require clinical escalation from those appropriate for watchful waiting.

Sixth, the depth of the developmental-plasticity argument as it pertains specifically to olfaction warrants further consideration. Olfaction-specific developmental evidence has recently strengthened the case for paediatric SSNOL as a clinically distinct entity. Murine work has demonstrated that early postnatal olfactory bulb activity is required for the proper maturation of entorhinal-hippocampal circuitry and later-life cognitive function (49). More strikingly, nasal chemosensation during the first postnatal week has been shown to drive a cross-modal critical window for somatosensory development, with neonatal odor deprivation producing enduring deficits in somatosensory processing in adulthood (50). These findings collectively suggest that paediatric SSNOL is unlikely to represent a simply attenuated version of the adult condition; the developmental window in which olfactory loss occurs may carry consequences that extend beyond the olfactory deficit itself, affecting downstream limbic, cognitive, and even other sensory systems. Prospective studies of paediatric SSNOL should therefore consider neurodevelopmental, cognitive, and multisensory endpoints alongside conventional olfactometric outcomes.

Accordingly, the SSNOL framework should be regarded as a hypothesis-generating model intended to stimulate further research, clinical discussion, and formal validation rather than a definitive classification system ready for immediate implementation.
